# TTR cardiac amyloidosis, polyneuropathy, and dementia at old age – bystander, cause or “smoking gun” guilty by association

**DOI:** 10.1002/alz.087146

**Published:** 2025-01-09

**Authors:** Rodica Elena Petrea, Katherine W. Turk, Mark D Knobel, Andreas Charidimou, Andrew E. Budson

**Affiliations:** ^1^ VA Boston Healthcare System, Jamaica Plain, MA USA; ^2^ Boston University Chobanian & Avedisian School of Medicine, Boston, MA USA; ^3^ Boston University School of Medicine, Boston, MA USA

## Abstract

**Background:**

Mixed dementia type – Alzheimer’s Disease (AD), cerebral amyloid angiopathy (CAA), and vascular ‐ is vastly recognized as a cause of dementia in older adults. Whereas CAA, primarily leptomeningeal, is a frequent complication in hereditary transthyretin cardiac amyloidosis (TTRCA), it is unusually reported in association with wild‐type TTR, with or without polyneuropathy. The knowledge of mixed dementia and wild‐type TTR association is even scarcer.

**Methods:**

Case Report. 81‐year‐old, Right‐handed man, with a 5‐year history of diagnosed wild‐type TTRCA and polyneuropathy, treated with Tafamidis, vascular risk factors, and remote history of benign head injuries, was seen in our Memory Disorders Clinic for evaluation of moderate dementia. MOCA score was 7/30 on the day of evaluation.

**Results:**

Our patient had 2 brain MRIs over 5‐year follow‐up. The first MRI was performed at initial memory complaints of mild cognitive impairment (MCI) concurrent with the diagnosis of cardiac amyloidosis and polyneuropathy. A second brain MRI was performed during prolonged transient neurological deficit ‐ aphasia with prominent word‐finding difficulties – accompanying delirium following septic knee arthritis surgery. The second MRI revealed foci of susceptibility weighted imaging (swi) artifact in line with superficial cerebral hemosiderosis in the left frontal lobe, and within the bilateral thalami, all new from the first MRI, which only revealed one focus of right periventricular white matter SWI microhemorrhage and moderate confluent white matter hyperintensities, more prominent in the left frontal and parietal lobes, progressed on the second MRI. The cognitive impairment (MCI to moderate dementia) and supporting MRI brain imaging evolved over 5 years and we indicate contributory components of probable CAA, vascular, and AD pathology.

**Conclusions:**

We report a rare association between probable AD, CAA, and vascular dementia, in a patient diagnosed with wild‐type TTRCA and polyneuropathy. We aim to increase awareness of dementia following the cardiac onset of wild TTRCA, possibly similar to the hereditary TTRCA. We inquire about the fortuitous or causative association of mixed dementia type and wild TTRCA, with or without polyneuropathy. We propose to study the intricately mixed etiology of cognitive impairment and its relationship to AD in wild TTRCA through a national registry.

